# Gene Expression Analysis Provides New Insights into the Mechanism of Intramuscular Fat Formation in Japanese Black Cattle

**DOI:** 10.3390/genes12081107

**Published:** 2021-07-21

**Authors:** Shuji Ueda, Mana Hosoda, Ken-ichi Yoshino, Minoru Yamanoue, Yasuhito Shirai

**Affiliations:** 1 Department of Agrobioscience, Graduate School of Agricultural Science, Kobe University, Kobe 657-8501, Japan; 203a422a@stu.kobe-u.ac.jp (M.H.); yamanoue@kobe-u.ac.jp (M.Y.); shirai@kobe-u.ac.jp (Y.S.); 2 Biosignal Research Center, Kobe University, Kobe 657-8501, Japan; kyoshino@kobe-u.ac.jp

**Keywords:** wagyu, adipocyte, RNA-seq, TGF-β, transcriptome, collagen, COL4A5, CPE, TNC, TAGLN

## Abstract

Japanese Black cattle (Japanese Wagyu) have a unique phenotype in which ectopic intramuscular fat accumulates in skeletal muscle, producing finely marbled beef. However, the mechanism of intramuscular fat formation in Japanese Black cattle remains unclear. To investigate the key genes involved in intramuscular fat accumulation, we comprehensively analyzed mRNA levels in subcutaneous and intramuscular fat tissues using RNA sequence (RNA-seq) analysis, which detected 27,606 genes. We identified eight key genes, namely carboxypeptidase E, tenascin C, transgelin, collagen type IV alpha 5 (COL4A5), cysteine and glycine-rich protein 2, PDZ, and LIM domain 3, phosphatase 1 regulatory inhibitor subunit 14A, and regulator of calcineurin 2. These genes were highly and specifically expressed in intramuscular fat tissue. Immunohistochemical analysis revealed a collagen network, including COL4A5, in the basement membrane around the intramuscular fat tissue. Moreover, pathway analysis revealed that, in intramuscular fat tissue, differentially expressed genes are related to cell adhesion, proliferation, and cancer pathways. Furthermore, pathway analysis showed that the transforming growth factor-β (TGF-β) and small GTPases regulators RASGRP3, ARHGEF26, ARHGAP10, ARHGAP24, and DLC were upregulated in intramuscular fat. Our study suggests that these genes are involved in intramuscular fat formation in Japanese Black cattle.

## 1. Introduction

Japanese Black cattle (i.e., Japanese Wagyu) are among the most expensive meats, characterized by excellent marbling, rich and sweet aroma (the so-called Wagyu beef aroma) [1,2]. Japanese Black cattle are pure Wagyu species that originate from pedigrees, such as Tajima, Kedaka, and Itozakura in Japan [3], whereas hybrid breeds with other cattle species are widespread all over the world. Marbling is a phenotype in which ectopic intramuscular fat accumulates in muscle tissue. In humans, excessive dietary lipid intake and age-related senescence lead to ectopic intramuscular fat in skeletal muscle. Fatty acid accumulation correlates with lipotoxicity-related cell dysfunction and is associated with sarcopenic obesity [4]. However, unlike cattle, humans accumulate intramuscular fat due to senescence and illness. Therefore, the marbling traits of Japanese Black cattle are considered a valuable research model for elucidating the molecular mechanism of ectopic intramuscular fat formation in various livestock, as well as in humans [5].

Intramuscular fat is thought to be formed by the myogenic transdifferentiation of myogenic stem cells [6] or a multi-step differentiation process from fibroblast-like preadipocytes to mature adipocytes [7]. The preadipocytes also regulate proliferation, differentiation, and lipogenesis with surrounding connective tissues through intercellular communication via paracrine factors, extracellular matrix (ECM), and cell-cell adhesion [8]. A genome-wide analysis has been conducted to study the genetic traits of intramuscular fat in livestock [9,10]; however, the molecular mechanism of intramuscular fat formation remains unknown. Intramuscular fat formation is a quantitative trait affected by environmental factors and genomic sequences. The difficulty in identifying key genes related to intramuscular fat accumulation is the requirement to consider environmental factors. Therefore, we performed a transcriptome analysis to attempt to elucidate this molecular mechanism. Recent improvements in the comprehensive analysis of large-scale RNA sequencing (RNA-seq) data have been generated, and several research institutes attempted to study mRNA expression profiles in cattle [11,12,13,14]. However, RNA-seq analysis has not been performed to report the transcriptome associated with pure Japanese Black cattle.

To search for the key genes involved in intramuscular fat accumulation, we performed comparative RNA-seq analyses of subcutaneous and intramuscular fat tissues from Japanese Black cattle, which exhibit low genetic and environmental variation. Additionally, we performed quantitative PCR (qPCR) analysis and tissue immunostaining of the identified characteristic genes to explore the molecular mechanism associated with intramuscular fat formation. Furthermore, we examined the molecular switches related to the signal transduction pathways involved in intramuscular fat formation using pathway analysis.

## 2. Materials and Methods

### 2.1. Reagents

The following antibodies used for immunostaining were purchased from Cosmo Bio Co., Ltd. (Tokyo, Japan): COL4A1 (CSB-PA001748), COL4A2 (CSB-PA007068), COL4A3 (CSB-PA007069), COL4A4 (PAC142HU08), COL4A5 (WLS-MAC141HU21), and COL4A6 (CSB-PA001750).

### 2.2. Japanese Black Cattle

In this study, we analyzed the intramuscular and subcutaneous fat of four Japanese Black cattle. The four cattle are of a similar breed. Their pedigree is a hybrid of the *Tajima*, *Kedaka*, and *Itozakura* strains, exhibiting excellent bodyweight growth [1,15,16]. The sampled cattle were selected from representative breeds (>27 months old; meat grade: ≥4; steers) produced in Japan. The cattle were fattened for 20 months on a common beef cattle diet consisting of grass hay plus compound feed (wheat bran, barley, feed corn, soybean meal, rice bran, minerals, vitamins, etc.). Immediately after slaughter, the sternocleidomastoid muscle of the cattle was collected by the technical staff of the Commercial Meat Processing Center (Kobe, Japan). This experiment does not include animal experimentation because the sampling was designed in the process of commercial distribution of beef with the cooperation of general livestock farmers.

### 2.3. RNA Preparation from Fat Tissue

Subcutaneous fat and intramuscular fat were collected and immersed in Nucleic acid preservation (NAP) buffer (19 mM EDTA, 18 mM trisodium citrate, and 3.8 M ammonium sulfate pH 5.2). After complete removal of fine connective tissue under a stereomicroscope, each tissue was cryopreserved at −80 °C.

Total RNA was purified using ~100 mg of fat tissue by the Maxwell RSC simply RNA tissue kit using a Maxwell RSC instrument (Promega K. K., Tokyo, Japan) according to manufacturer instructions. The concentration of the obtained RNA was measured using a NanoDrop spectrophotometer (Thermo Fisher Scientific K.K., Tokyo, Japan). RNA samples were quality checked by the Agilent 2200 TapeStation (Agilent Technologies Japan, Tokyo, Japan), and high-purity samples with a degradation index RNA integrity number equivalent ≥ 7.6 were subjected to an RNA-seq analysis.

### 2.4. Preparation of Sequence Library

Purified RNA (10 ng) was amplified by PCR amplification (7 cycles) using the Clontech SMART-Seq v4 Ultra Low Input RNA kit (Takara Bio, Kusatsu, Japan) for construction and sequencing of the total RNA-seq library. The PCR product was purified by the magnetic bead method using AMPure XP (Beckman Coulter, Tokyo, Japan). Double-stranded cDNA (0.2 ng) was generated and barcoded by PCR amplification (11 cycles) using the Nextera XT DNA Library Prep kit (Illumina K.K., Tokyo, Japan). The sequence library was validated using a fragment analyzer (Agilent Technologies, Tokyo, Japan)

### 2.5. RNA-seq Analysis

The sequence library was sequenced on the Illumina NovaSeq 6000 with a NovaSeq 6000 S4 reagent kit and NovaSeq Xp 4-Lane kit (Illumina K.K.). Analyses were performed according to a sequence read length of 150 bp via paired-end sequencing using NovaSeq control software (v1.6.0), RTA (v3.4.4), and bcl2fastq2 (v2.20) from Illumina K.K..

The Genedata Profiler Genome (v.13.0.11; Genedata K.K., Tokyo, Japan) was used for sequence analysis. The read sequence was mapped onto the genome sequence using the mapping software STAR (v.2.6.0c) annotated from the obtained genome position, and the expression level was calculated for each gene and transcript. Data were analyzed using the bovine genome sequence ARS-UCD1.2 (GCA 002263795.2) and gene databases (*Bos taurus*; ARS-UCD1.2.dna.toplevel.fa.gz (ftp://ftp.ensembl.org/pub/release-99/fasta/homo_sapiens/dna/, accessed on 13 March 2020) and ARS-UCD1.2.99.gtf.gz (ftp://ftp.ensembl.org/pub/release-99/gtf/bos_taurus/, accessed on 13 March 2020).

### 2.6. Gene Expression Analysis

Differentially expressed genes (DEGs) were determined by comparing the difference between the log_2_ transcripts per million (TPM) values of intramuscular and subcutaneous fat tissue, which was statistically evaluated with a *t*-test (significance: *p* < 0.05). Clustering analysis was performed using a 1625 DEGs MultiExperiment Viewer (Mev v.4.9, https://sourceforge.net/projects/mev-tm4/, accessed on 12 April 2021). A Gene Ontology (GO) analysis and a Kyoto Encyclopedia of Genes and Genomes (KEGG) analysis were performed on 1625 DEGs using the Database for Annotation, Visualization, and Integrated Discovery (DAVID v.6.8, http://www.genome.jp/kegg/, accessed on 12 April 2021).

### 2.7. qPCR Analysis

cDNA libraries were constructed using ReverTra Ace qPCR RT master mix with gDNA Remover (Toyobo, Osaka, Japan). qPCR was performed using the One-step TB Green Primescript RT-PCR kit II (Takara Bio) and a Dice Real-Time System Lite thermocycler (Takara Bio) according to manufacturer instructions, with bovine β-tubulin used as an internal standard. The thermocycling conditions were as follows: heating at 95 °C for 30 s, followed by 40 to 47 cycles of denaturation at 95 °C for 15 s, annealing for 60 s at the indicated temperatures for each primer (Figure S1), and extension at 60 °C for 30 s.

### 2.8. Tissue Staining with Paraffin-Embedded Sections

Musculus longissimus lumborum was paraffin-embedded and sectioned at a 5-μm thickness, as described previously [17]. Paraffin sections were deparaffinized with a lemosol solution (Fujifilm Wako Chemicals, Osaka, Japan). Collagen fiber staining was performed using a Picro-Sirius Red stain kit (ScyTek Laboratories, Logan, UT, USA).

For tissue immunostaining, deparaffinized sections were activated with an antigen-retrieval solution (Histo VT one solution; Nacalai Tesque, Kyoto, Japan) at 90 °C for 20 min. After permeabilization with 0.3% Triton-X in PBS (−) for 30 min and inactivation of endogenous peroxidase with 0.3% H_2_O_2_ solution for 30 min, the sections were treated overnight with an antibody diluted with Can Get Signal immunostaining solution A (Toyobo). The bound antibody was visualized using Histofine Max-Po (Nichirei Biosciences, Tokyo, Japan) and ImmPACT DAB (Vector Laboratories, Burlingame, CA, USA). Counterstaining was performed with Mayer’s hematoxylin solution (Fujifilm Wako Chemicals), and image analysis was performed using an all-in-one microscope system (BZ8000; Keyence, Osaka, Japan).

### 2.9. Statistical Analysis

The density of protein bands was measured using an image analysis software (Image J, National Institutes of Health, Bethesda, MD, USA). A multivariate data analysis of the Orthogonal Projections to Latent Structures Discriminant Analysis (OPLS-DA) was performed using the SIMCA14 software (Inforcom, Tokyo, Japan) [15,18]. Statistical significance was determined at a *p* < 0.05 according to Student’s *t*-test with JMP12 (SAS Institute Japan, Tokyo, Japan) and Excel 2019 (Microsoft Japan, Tokyo, Japan). The q-values for the GO and KEGG analyses were performed using DAVID v.6.8 according to the manual.

## 3. Results

### 3.1. RNA-seq Analysis of Subcutaneous Fat and Intramuscular Fat

**Figure 1:**
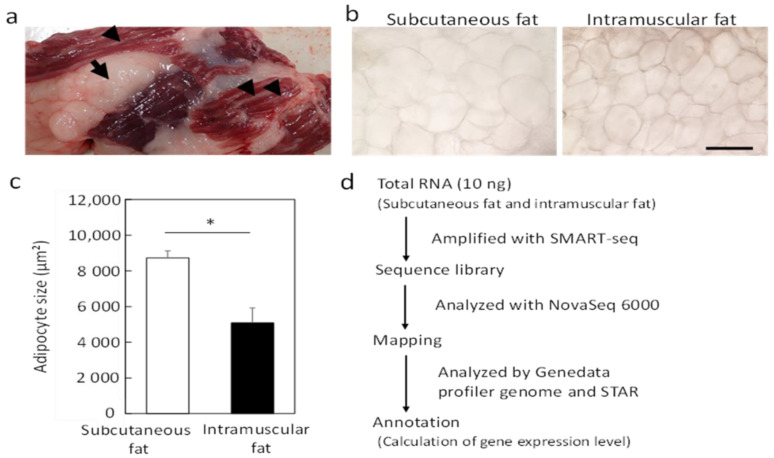
Overview of fat samples and RNA-seq analysis. (**a**) Image of subcutaneous fat and intramuscular fat in the sternocleidomastoid muscle. Arrows indicate subcutaneous fat, and arrowheads indicate intramuscular fat. (**b**) Phase-contrast microscopic images of each adipocyte. Scale bar, 100 μm. (**c**) Mean value of the cross-sectional area of adipocytes in each fat tissue (± standard deviation, * *p* < 0.05). (**d**) Summary of RNA-seq analysis. We analyzed the intramuscular and subcutaneous fat of four Japanese Black cattle by RNA-seq analysis.

During the analysis of a small amount of intramuscular fat samples (like marble in muscle) collected from Japanese Black cattle, we also collected subcutaneous fat from the same cattle to compare with intramuscular fat (Figure 1a). We found no difference in the appearance of adipocytes between subcutaneous and intramuscular fat tissues, although the size of adipocytes was significantly smaller (*p* < 0.05) in intramuscular fat (Figure 1b,c). RNA-seq analysis following the establishment of a sequence library revealed a mean total read count of 149,293,058 reads for subcutaneous fat and 167,613,200 reads for intramuscular fat. The read counts mapped to the genetic database ere 131,204,412 for subcutaneous fat (87.9% of total reads) and 147,205,196 reads for intramuscular fat (87.8%). The data had a primary map count of 131,271,893 reads, a secondary map count of 7,932,911 reads, and an average insert size of 224 bp.

**Figure 2:**
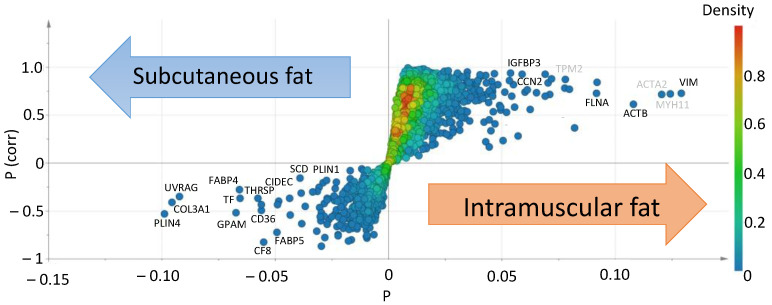
Comparison of gene expression between intramuscular fat and subcutaneous fat. S-shaped plot for the OPLS-DA model, which was calculated based on RNA-seq data. The horizontal axis p is a variable that indicates the measure of correlation obtained with OPLS-DA analysis. The vertical axis p (corr) is an abbreviation for p scaled by the correlation coefficient, which is the loading of each variable scaled as a correlation coefficient and standardized in the range of −1 to +1. P (corr) indicates the relationship between intramuscular fat or subcutaneous fat, either positive or negative. The plot shows variable genes in subcutaneous fat (left) and intramuscular fat (right). The names of highly expressed genes in adipocytes are in black, and those of highly expressed genes in muscle fibers are in gray. R2X = 0.228, and scaling sets par type. The scores for the OPLS-DA model were R2 (cum) = 0.969 and Q2 (cum) = 0.788. The different colors in the plot indicate differences in density. The density bar on the right shows the color of the degree of density of the plot.

After extracting expressed genes from the primary analysis data, a total of 27,606 genes were detected. Gene expression levels were normalized as TPM, and false-positive genes were removed. To comprehensively view gene expression in each fat tissue, we performed a multivariate analysis of the data and displayed the genes as a visual plot (Figure 2). Actin alpha 2 (ACTA2), tropomyosin 2 (TPM2), and myosin heavy chain 11 (MYH11) are typical genes of muscle fibers, predicted as contaminants, and removed from the analytical data.

**Figure 3:**
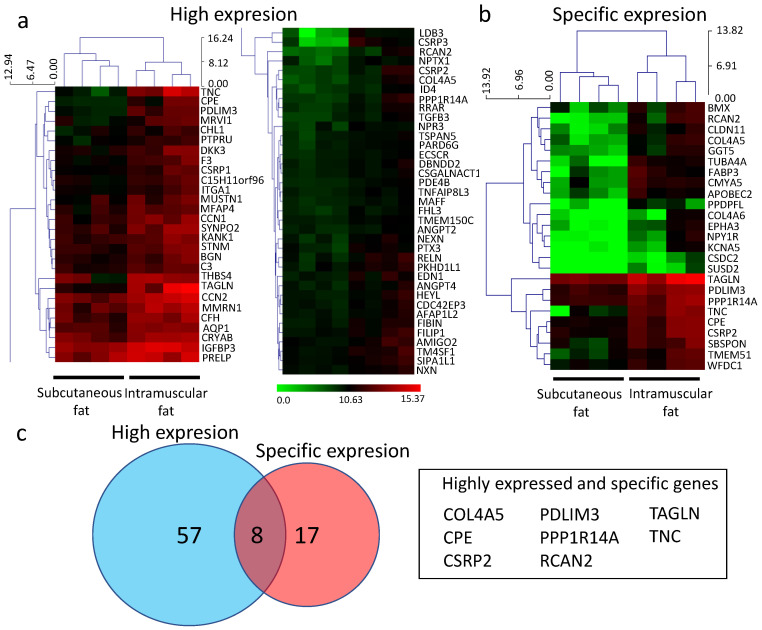
Analysis of gene expression in subcutaneous and intramuscular fat tissues. (**a**) Heat map showing 65 genes highly expressed in intramuscular fat (count value ≥ 1000, the expression ratio of int/sub ≥ 2; *p* < 0.05). (**b**) Heat map showing 25 genes specifically expressed in intramuscular fat and with a low expression level (count value ≥ 16, expression ratio of int/sub ≥ 4; *p* < 0.05). The expression ratio is shown with upregulated genes in red and downregulated genes in green. (**c**) Venn diagram showing the number of highly and specifically expressed genes in intramuscular fat.

We then compared gene expression between intramuscular fat and subcutaneous fat. First, the TPM values were log-transformed and then compared between the two groups of subcutaneous fat and intramuscular fat, and the genes that obtained positive or negative significant differences by *t*-test were extracted as DEGs. The extraction of DEGs revealed 1625 genes expressed at significantly higher levels in intramuscular fat than in subcutaneous fat and 130 genes showing higher expression in subcutaneous fat than intramuscular fat (*p* < 0.05). We first investigated genes showing significantly higher expression in intramuscular fat than in subcutaneous fat. Of the 1625 DEGs in intramuscular fat, 65 were identified as highly expressed genes (count value ≥ 1000, the expression ratio of int/sub ≥ 2; *p* < 0.05; Figure 3a) and 25 as specifically expressed genes (count value ≥ 16, expression ratio of int/sub ≥ 4; *p* < 0.05; Figure 3b). Of these, we identified eight new genes, namely carboxypeptidase E (CPE), tenascin C (TNC), transgelin (TAGLN), collagen type IV alpha 5 (COL4A5), cysteine and glycine-rich protein 2 (CSRP2), PDZ and LIM domain 3 (PDLIM3), phosphatase 1 regulatory inhibitor subunit 14A (PPP1R14A), and regulator of calcineurin 2 (RCAN2), as highly and specifically expressed genes (Figure 3c). The 65 highly expressed genes and 25 specifically expressed genes are listed in Figure S2, and the top 15 highly expressed genes in intramuscular fat are shown in Figure S3.

### 3.2. Verification of Characteristic Genes Expressed in Intramuscular Fat

**Figure 4:**
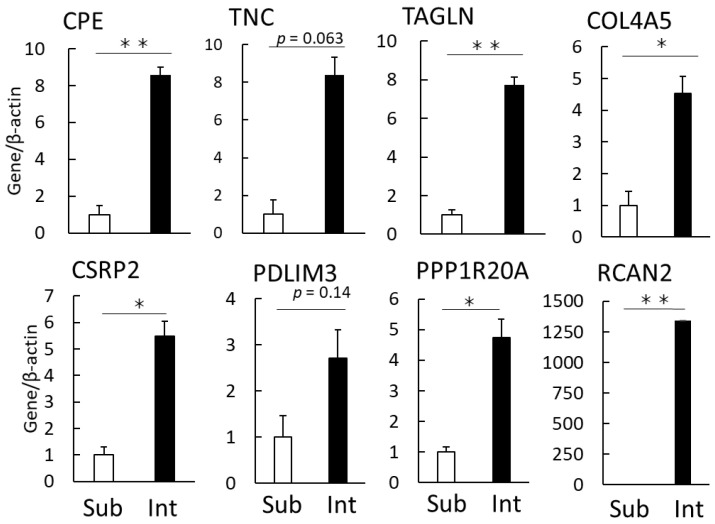
Verification of eight genes highly expressed in intramuscular fat via qPCR. qPCR was performed using cDNA synthesized from the RNA of subcutaneous fat (Sub) and intramuscular fat (Intra). Graphs show the mean values relative to the expression levels in Sub. Data represent the mean ± standard error (*n* = 3). ** *p* < 0.01, * *p* < 0.05.

We then verified the reliability of the expression data using qPCR. We verified the eight genes identified as highly expressed in intramuscular fat, and significant differences were confirmed for six genes (Figure 4). The reproducibility of the gene expression was confirmed for each gene.

### 3.3. Expression Analysis of the COL4 Isoform

**Figure 5:**
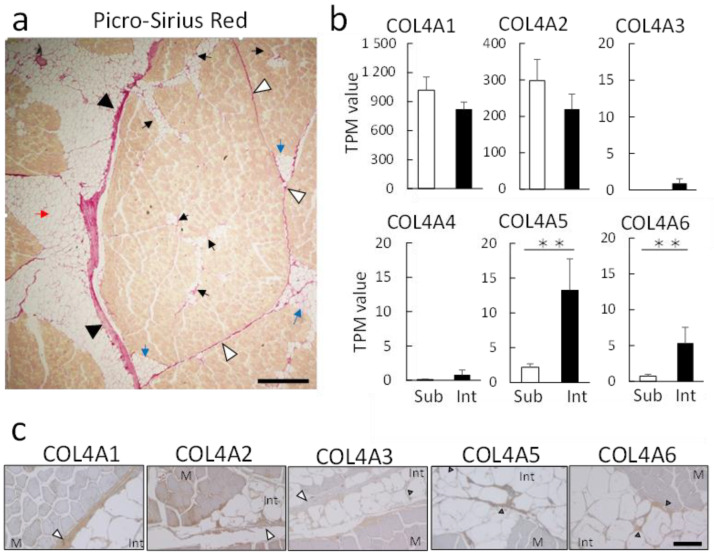
A network of COL4A in the basement membrane of intramuscular fat. (**a**) Paraffin-embedded sections were prepared from the musculus longissimus lumborum of Japanese Black cattle. Picro-Sirius Red staining showing collagen (red) and muscle (yellow) fibers. Arrow indicates intramuscular adipocytes. The red arrow indicates the large intramuscular fat around the epimysium. Blue arrows indicate relatively thin intramuscular fat around the perimysium. Black arrows indicate the micro-intramuscular fat around the endomysium in the muscle bundle. Black arrowheads indicate the epimysium, white arrowheads indicate the perimysium, and gray arrowheads indicate the thickened basement membrane of the endomysium. Size bar, 500 μm. (**b**) Comparative analysis of COL4A expression by TPM values. Graphs show the TPM values obtained by RNA-seq analysis. Data represent the mean ± standard error (*n* = 3). ** *p* < 0.01, (**c**) Tissue immunostaining for COL4A family proteins in paraffin sections of muscle tissue from Japanese Black cattle. DAB staining (brown) was performed using primary antibodies targeting specific isoforms, with hematoxylin used for counterstaining. White arrowheads indicate perimysium, and gray arrowheads indicate endomysium. Size bar, 100 μm. Int, intramuscular adipocytes; M, muscle tissue.

Intramuscular fat comprises adipocytes that ectopically proliferate and differentiate around the epimysium, perimysium, and endomysium [19] (Figure 5a). In the present study, we focused on COL4A5. COL4A has six different isoforms, the chains of which form heterotrimers to construct a complex collagen network in the basement membrane [20]. Comparison of the expression of the six isoforms revealed COL4A5 and COL4A6 as showing the highest expression in intramuscular fat relative to subcutaneous fat (Figure 5b), with this confirmed by qPCR (Figure S4). We then confirmed COL4A isoform expression in the basement membrane around intramuscular fat by tissue immunostaining. Unlike other collagens, COL4A is not expressed in muscle tissue. We observed that COL4A isoforms were unevenly distributed in the perimysium and endomysium around the muscle fibers (Figure 5c). COL4A5 and COL4A6 were only distributed in the thickened basement membrane around adipocytes in the endomysium. By contrast, COL4A1 and COL4A2 abundantly localized in the perimysium, whereas COL4A3 and COL4A4 (data not shown) were detected at low levels in the endomysium.

### 3.4. Pathway Analysis of Differentially Expressed Genes

**Figure 6:**
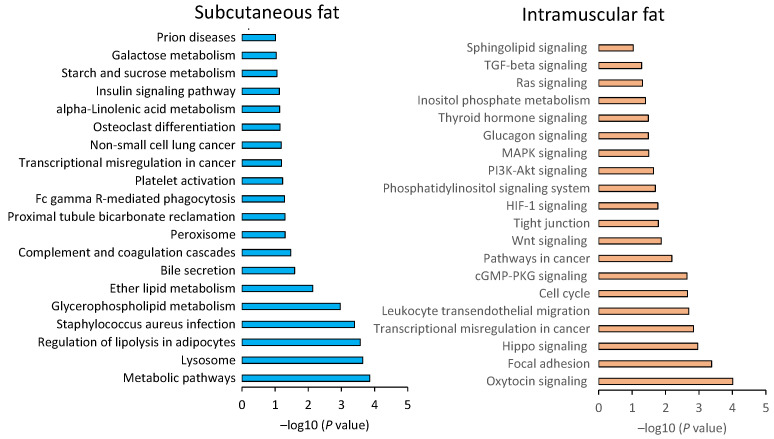
Pathway analysis of DEGs in intramuscular fat. KEGG enrichment analysis revealed significant pathways for the 1625 DEGs in subcutaneous fat and intramuscular fat. The *X*-axis represents the *p*-value obtained using the Benjamini–Hochberg test.

We then evaluated the intracellular functions of DEGs using a KEGG enrichment analysis and a GO analysis. KEGG results showed the characteristic pathways for intramuscular and subcutaneous fat (Figure 6). DEGs in intramuscular fat were significantly associated with 20 KEGG pathways, including focal adhesions (34 genes; *p* = 0.0004), pathways in cancer (50 genes; *p* = 0.0065), PI3K-Akt signaling (42 genes; *p* = 0.0231), MAPK signaling (32 genes; *p* = 0.0316), Ras signaling (29 genes; *p* = 0.0493), and transforming growth factor (TGF)-β signaling (13 genes; *p* = 0.0518). By contrast, DEGs in subcutaneous fat were associated with cellular metabolism pathways, including metabolic pathways (38 genes; *p* = 0.0001), lysosomes (10 genes; *p* = 0.0002), and the regulation of adipocyte lipolysis (7 genes; *p* = 0.0002).

**Figure 7:**
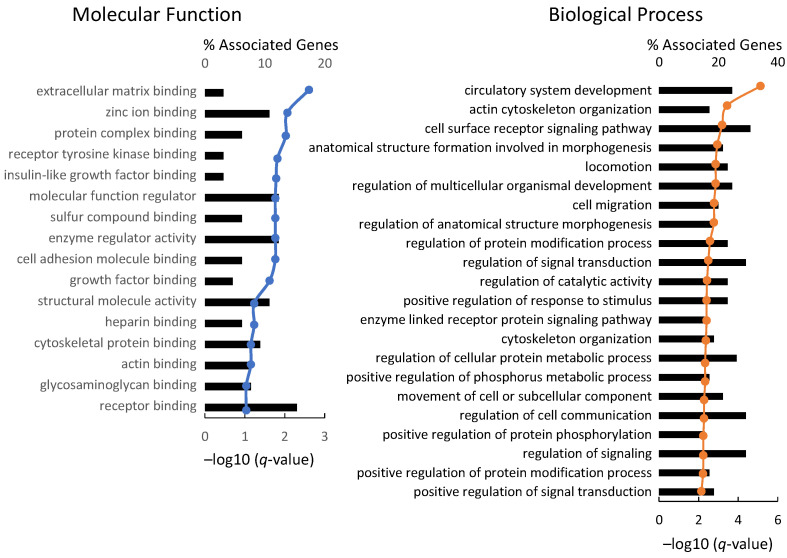
Functional analysis of DEGs in intramuscular fat. GO analysis revealed significant functions for 1625 DEGs in intramuscular fat. The graph shows GO terms in the categories of biological processes and molecular function. The X-axes represent the percentage of genes that matched the GO term. The clustered line plots represent the q-values obtained by Fisher’s exact test and relative to the other terms.

To further narrow down the genes involved in intramuscular fat formation, we analyzed the cellular functions of these genes using GO (Figure 7). A total of 1625 DEGs were significantly associated with ECM binding (3.8%; q-value, 2.60) and zinc ion binding (10.8%; q-value, 2.05) as a molecular function. Intramuscular fat was also associated with circulatory system development (24.6%; q-value, 5.12), actin cytoskeleton organization (16.9%; q-value, 3.44), cell surface receptor signaling (30.7%; q-value, 3.19), and cell migration (20.0%; q-value, 2.78) among biological processes. GO analysis revealed that the DEGs of intramuscular fat were associated with ECM binding, cell-cell adhesion, angioplasty, and receptor signal. These findings suggest that the surrounding environment influences intramuscular fat accumulation. The intracellular function of intramuscular adipocytes resembles that of infiltrating cells and tumors in response to the microenvironment [21]. This unique cellular environment is seemingly involved in intramuscular fat accumulation.

### 3.5. Analysis of COL4-Related Cellular Signals in Intramuscular Fat

We hypothesized that the ECM of the basement membrane contributes to intramuscular fat formation. TGF-β, integrins, and cell migration factors represent ECM-related signals (Table 1). TGF-β is a growth factor that induces COL4A secretion in connective tissue [22]. TGFB2 and TGFB3 were highly expressed in intramuscular fat tissue compared to subcutaneous fat tissue, whereas TGFB1 was expressed at a lower level. SMAD, a major transcription factor that functions in the canonical pathway of TGF-β, showed similar expression levels between subcutaneous and intramuscular fat tissues. However, the expression levels of SMAD7, CHRD, and TGIF1, which are regulators of TGF-β signaling, are significantly differed between tissues. Furthermore, heterodimers, namely ITGA2, ITGB1, and ITGB6, were significantly highly expressed in intramuscular fat compared to subcutaneous fat tissue. The expression of heterodimers, integrin α (ITGA) and integrin β (ITGB), ITGA2, ITGB1, and ITGB6, was significantly higher in intramuscular compared to subcutaneous fat tissue. By contrast, ITGA6, which encodes the major ITGA in adipocytes [23], was highly expressed in subcutaneous fat tissue. Concerning cell migration factors, CXCL5, FGF9, and FGF13 showed significantly higher expression in intramuscular fat relative to subcutaneous fat, whereas CXCR4, which is a receptor for CXCL5, showed similar expression levels between subcutaneous and intramuscular fat tissues.

We then evaluated the Ras signal pathway involving Ras protein, which at acts as a molecular switch to control various signal transduction pathways. GDP/GTP-exchange factor (GEF), GTPase activating factor (GAP), and effectors are listed as regulators of small GTPases [24]. Among GEFs, RASGRP3 and ARHGEF26 were highly expressed in intramuscular fat relative to subcutaneous fat. For GAP, ARHGAP10 and ARHGEF24 were highly expressed in intramuscular fat, as were RASSF9 and RASSF10, which are effector molecules for Ras GTPase.

## 4. Discussion

Japanese Black cattle are not crossed with other cattle breeds, and their pedigree is strictly controlled; therefore, the use of Japanese Black cattle in research introduces low genetic variation. We previously analyzed the metabolite [15] and lipid composition [18] of intramuscular fat in Japanese Black cattle. In this study, we performed RNA-seq analysis to explore the key genes involved in the intramuscular fat accumulation of Japanese Black cattle. Previous studies indicated that comparisons between different cattle breeds tend to detect DEGs related to popular adipocyte functions, such as lipid metabolism and adipogenesis [12]. In the present study, we focused on intramuscular fat formation-related genes. Therefore, to avoid bias, we collected intramuscular and subcutaneous fat tissues from the same cattle.

Microscopic observation showed little contamination of other tissues (Figure 1b). Intramuscular fat is known to comprise a smaller portion of adipocytes than subcutaneous fat [25]. We confirmed that the intramuscular fat used in this analysis comprised a smaller portion of adipocytes than subcutaneous fat (Figure 1c). RNA-seq analysis was performed on a small sample (100 mg) to avoid contamination with other connective tissues (Figure 1d). A higher number of DEGs was detected in intramuscular than in subcutaneous fat tissues (Figure 2). The muscle-derived myosin and actin genes were detected by RNA-seq. These genes might be derived from a fragment of muscle tissue attached to the sample or intramuscular fat-specific cytoskeletal proteins [16]. To render our search more comprehensive, we explored characteristic genes of intramuscular fat using two search criteria for genes, high expression and specificity. Of the identified genes, we have identified eight novel genes highly and specifically expressed in intramuscular fat tissues from Japanese Black cattle (Figure 3). These eight genes were validated as highly expressed in intramuscular fat tissue by quantitative PCR assays (Figure 4).

The role of these eight genes in intramuscular fat formation was inferred from their respective functions. TNC is a high molecular weight glycoprotein that plays an ECM to stabilize the basement membrane with collagen. Interestingly, TNC is reportedly induced by obesity-related inflammatory responses in the adipose tissue [26] and tumor microenvironment [27]. Tenascin has four isoforms, TNC, TNR, TNXB, and TNW, in cattle. In our RNA-seq data, only TNC was highly detected in intramuscular fat. TAGLN is a TGF-β inducible gene that functions as an actin cross-linking protein [28]. TAGLN also promotes cell migration and adipocytic differentiation of human bone marrow-derived stromal stem cells [29]. CPE acts as an exopeptidase and is involved in the biosynthesis of neuropeptides and peptide hormones in endocrine tissues and the nervous system. Deficiency in CPE activity has been linked to diabetes, obesity, and reduced learning ability [30]. Genetic analyses highlighted that single nucleotide polymorphisms in CPE are associated with beef quality [31]. Of these eight genes, CPE, TNC, and TAGLN have been detected in the intramuscular fat of other cattle [32]. These three genes are presumed to be essentially expressed in intramuscular fat regardless of cattle pedigree.

CSRP2 contains three isoforms (CSRP1, CSRP2, CSRP3). These CSRPs act as transcription factors in the nucleus and promote cell differentiation in various cell types. CSRP2 expression is reportedly induced by TGF-β [33], an essential gene for epithelial-mesenchymal conversion [28]. Although CSRP1, CSRP2, and CSRP3 were highly expressed in intramuscular fat tissue (Figure S2), their detailed function is unknown. RCAN2 is involved in muscle differentiation and cancer progression [34]. PPP1R14A is a typically upregulated gene in obesity-related hypertension [35]. PDLIM3 are sarcomere-related proteins. Of note, PDLIM3 regulates cell-cell adhesion and migration and promotes malignant metastasis [36]. Unlike other collagens, COL4A is a unique collagen isoform in the basement membrane [37]. COL4A5 forms supramolecular networks with COL4A [20].

Tissue immunostaining showed that COL4A5 colocalized with COL4A6 in the thickened basement membrane of the endomysium (Figure 4). Based on their expression patterns (Figure 5b), COL4A1 and COL4A2 seemed to contribute to both subcutaneous and intramuscular fat accumulation, whereas COL4A5 may be involved in the “micro-intramuscular fat” (Figure 5a). Recently, the specific expression of COL4A5 in the tumor microenvironment has been reported [38]. COL4A5 expression in the basement membrane may involve the infiltration and proliferation of ectopic cells, such as intramuscular fat and tumors.

Next, we investigated the intracellular functions of DEGs in each fat tissue. KEGG analyses revealed that intramuscular fat is associated with gene pathways related to cell migration and proliferation (Figure 6). Notably, PI3K-Akt, MAPK, and Ras signaling pathway upregulation suggest the activation of downstream membrane receptors and ECM binding in intramuscular fat tissues. The cellular function associated with intramuscular fat was consistent with the GO analysis results (Figure 7).

From these results, we focused on the ECM and compared the TPM values of genes related to TGF-β, cell adhesion, and cell migration factors (Table 1). TGF-β regulates gene expression by SMAD-mediated signal transduction [39]. TGF-β reportedly induces CPE, TNC, TAGLN, CSRP2, and COL4A5 expression [28,33,40]. Therefore, it is suggested that TGF-β/SMAD pathway-mediated promotion of gene transcription might underly intramuscular fat formation in Japanese Black cattle. Furthermore, ITGA6 and ITGB1, the subunits of integrin α6β1 that specifically bind to the COL4A5 heterotrimer [20], were increased in intramuscular fat. The expression of cell migration factors CXCL5, FGF9, and FGF13, was also increased in intramuscular fat tissue (Table 1). Therefore, in the intramuscular fat tissue, increased cell adhesion to the basement membrane may promote preadipocyte cell migration by increasing ITGB1, CXCL5, FGF9, and FGF13 expression.

Surprisingly, cancer-related pathways were significantly associated with intramuscular fat tissue (Figure 6), including TGF-β, Wnt-1, Ras, and Rho signaling-related genes (Figure S5). The TGF-β is known as a non-canonical pathway. This pathway activates Ras/MAPK signaling and the Rho family signaling of small GTPase in a SMAD-independent manner [40]. These small GTPase-mediated TGF-β signaling reportedly promote cell migration and differentiation in various inflammatory tissues and tumors [39]. These findings suggest that the cell function specific to intramuscular fat accumulation might be mediated by TGF-β. Therefore, TGF-β is considered a pivotal gene in intramuscular fat formation in Japanese Black cattle. Comparison between two groups by DEGseq2 is shown in (Figure S6).

The TPM levels did not differ between intramuscular and subcutaneous fat tissues in the Ras and Rho family of small GTPase. Many previous studies have shown that small GTPase activity is specifically regulated during signal transduction [24,41]. Therefore, we compared the expression of key regulators between intramuscular and subcutaneous fat tissues. RNA-seq analyses detected 66 genes as regulators of the Ras or Rho family of small GTPases. Of these, RASGRP3 and ARHGEF26 were significantly more highly expressed in intramuscular fat tissue than subcutaneous fat tissue (Table 1). RASGRP3 activates the Ras signals and promotes invasion and cell migration [42]. ARHGEF26 activates the Rho family of RhoG and also regulates cancer cell migration [43]. Besides, ARHGAP10, ARHGAP24 [44], and DLC3, which act as GAPs for the Rho family [45], were highly expressed in intramuscular fat. Moreover, ARHGEF33 expression was significantly lower in intramuscular fat tissue than subcutaneous fat tissue. Differential expression of GEFs and GAPs has been widely reported in tumors. These regulators are reportedly sufficient to regulate small GTPase activities by altering their expression levels [46]. These regulators may also regulate the small GTPase activities required for the proliferation, differentiation and maintenance of adipocytes around the basement membrane of the endomysium that compose the intramuscular fat.

## 5. Conclusions

In this study, our RNA-seq analysis led to identifying eight genes highly and specifically expressed in the intramuscular fat of Japanese Black cattle. Among them, COL4A5 was found to be highly expressed in the basement membrane around intramuscular fat. Pathway analysis also revealed that cell adhesion, cell proliferation, and cancer pathways are involved in intramuscular fat formation. In addition, the relationship between TGF-β signaling and the regulators of Ras and Rho families are seemingly involved in intramuscular fat accumulation. These genes might be used as molecular markers to better understand intramuscular fat formation [15].

## Figures and Tables

**Table 1:** List of differentially expressed genes related to the extracellular matrix.

No.	Gene	Name	Mean of ^a^ TPM Value		Ratio	*t*-Test
			Subcutaneous Fat	Intramuscular Fat	(Int/Sub)	*(p* Vales)
TGF-β signal						
1	TGFB2	Transforming growth factor beta 2	24.90	30.87	1.24	0.010 *
2	TGFB3	Transforming growth factor beta 3	5.34	17.70	3.32	0.038 *
3	TGIF1	TGFB induced factor homeobox	26.85	36.77	1.37	0.016 *
4	SMAD7	SMAD family member 7	3.69	6.27	1.70	0.011 *
5	CHRD	Chordin	4.68	6.82	1.46	0.011 *
6	TGFBR1	Transforming growth factor beta receptor 1	44.31	50.05	1.13	0.113
7	TGFB1	Transforming growth factor beta 1	98.20	61.95	0.63	0.056
8	SMAD2	SMAD family member 2	78.02	85.55	1.10	0.289
9	SMAD3	SMAD family member 3	15.73	16.63	1.06	0.826
10	SMAD4	SMAD family member 4	47.21	51.80	1.10	0.371
11	SMAD6	SMAD family member 6	6.05	8.37	1.38	0.209
Integrin						
12	ITGA2	Integrin subunit alpha 2	0.56	1.32	2.36	0.044 *
13	ITGB1	Integrin subunit beta 1	528.35	710.96	1.35	0.029 *
14	ITGB6	Integrin subunit beta 6	0.02	0.45	25.52	0.017 *
15	ITGA6	Integrin subunit alpha 6	211.64	186.69	0.88	0.521
Cell migration factor						
16	CXCL5	Chemokine (C-X-C motif) ligand 5	0.52	2.19	4.23	0.046 *
17	CXCR4	C-X-C motif chemokine receptor 4	9.56	22.76	2.38	0.087
18	FGF9	Fibroblast growth factor 9	1.89	3.11	1.65	0.036 *
19	FGF13	Fibroblast growth factor 13	1.07	1.88	1.75	0.040 *
Regulators of small GTPase						
20	RASGRP3	Ras guanyl releasing protein 3	9.02	16.91	1.87	0.003 **
21	ARHGEF26	Rho guanine nucleotide exchange factor 26	3.96	6.18	1.56	0.022 *
22	ARHGAP10	Rho GTPase activating protein 10	27.56	47.61	1.73	0.025 *
23	ARHGAP24	Rho GTPase activating protein 24	10.02	14.49	1.45	0.046 *
24	DLC3	Deleted in liver cancer 3 (STARD8)	9.32	13.59	1.46	0.041 *

^a^ Data represent the mean transcripts per million (TPM) values (*n* = 4) relative to the expression levels between intramuscular fat and subcutaneous fat. * *p* < 0.05, *t*-test. ** *p* < 0.01, *t*-test.

## Data Availability

The data presented in this study are contained in the article.

## Supplementary Materials

The following are available online at https://www.mdpi.com/article/10.3390/genes12081107/s1. Figure S1: List of primer sequences used for qPCR. Figure S2: List of 65 highly expressed genes and 25 genes specifically expressed in intramuscular fat. Figure S3 List of top 15 genes showing elevated expression in intramuscular fat and subcutaneous fat. Figure S4: Expression of COL4A isoforms by qPCR. Figure S5: List of pathway analyses. Figure S6: Comparison between two groups by DEGseq2 package.
