# Supplementary material for: Gene Expression Analysis Provides New Insights into the Mechanism of Intramuscular Fat Formation in Japanese Black Cattle
Source: Genes (Basel). 2021 Jul 21;12(8):1107. doi: 10.3390/genes12081107 (PMC8391117; doi:10.3390/genes12081107)
Supplement: Supplementary file 1 [file genes-12-01107-s001.zip › genes-1241299-supplementary.pdf]

Supplementary data

Gene	NCBI number		Sequence
TNC	NM_001372074.1	F	TTTGACAGCTTCCTAGTCAC
		R	CTCATAGCCAATGCCAGTTA
GGT5	NM_001191219.3	F	CACCAGCACCATCAACAC
		R	CTGTGCTCACCGGGC
TAGLN	NM_001046149.1	F	TCGAGAAGAAGTATGACGAG
		R	AGGATACAGGCTATTGACCA
CPE	NM_173903.4	F	GATTTCAATTACCTCAGCAGC
		R	AAATCCTTTAACTCCTCGGTG
NPY1R	NM_001045898.1	F	TGAGATCGTTCAGAAGTGAC
		R	GGGAACTGGTTTTCCACTTA
TMEM51	NM_001098975.1	F	CAAGACCTGAGGATGCATAA
		R	AGAAGGAACAGAACATCTCAG
COL4A5	XM_002699860.4	F	TGCTGTATTCTCCAAGACAC
		R	ACAGGGAAGAAAATCGTCAA
CSRP2	NM_001038183.1	F	GTGGTCCTCCTTCCGATTT
		R	ATCTAAATTCTTCCTGCAAACC
PDLIM3	NM_001034646.1	F	TCAACTTAGAATCAGAACCACA
		R	ATTGCTGGTTGAATAGAGCC
PPP1R14A	NM_001193070.2	F	CAAGTATGACCGGCGAGAG
		R	CCTCTTCACTCTCTAATTCCAA
RCAN2	NM_001015632.2	F	TGTCATTGCCATCAGAGAG
		R	AAGAACCTTCCAGGTAGATG
BMX	NM_001192707.2	F	ACAACACCTCAAAGATGTCA
		R	TTGTGACCTGGAGATGTTAC

Gene	NCBI number		
COL4A1	NM_001166511.3	F	AAATAGGTTTCCCAGGACAG
		R	AGCCGTATGTCGAAGTAGAT
COL4A2	XM_025000171.1	F	GGTACAGTCTGCTCTACTTC
		R	GTTACGGCTGGCATAGTAG
COL4A3	NM_001166529.2	F	CCGAGCCAGTCCATTTATAG
		R	TTTCTAACTCCCCAGCTTTC
COL4A4	XM_024981732.1	F	GCTGCCTAGAGGATTTCAG
		R	CTTTCAAGGTGTCCGGTAAT
COL4A6	XM_005227897.4	F	CCCTAGTGGTTTATCAGGGT
		R	GAATTCCATTGATGCCAAGAA

Figure S1. List of primer sequences for qPCR.

Gene	Mean of TPM value		Ratio	*t*-test
	Subcutaneous fat	Intramuscular fat	Intra/Sub	(*P* vales)
AFAP1L2	12.30	22.93	1.86	0.042
AMIGO2	20.33	42.43	2.09	0.034
ANGPT2	5.90	10.88	1.85	0.034
ANGPT4	13.97	30.10	2.15	0.041
AQP1	84.76	163.02	1.92	0.000
BGN	56.30	125.10	2.22	0.049
C15H11orf96	67.21	149.38	2.22	0.021
C3	22.63	41.67	1.84	0.005
CCN1	65.24	172.69	2.65	0.007
CCN2	132.65	356.31	2.69	0.007
CDC42EP3	19.15	38.04	1.99	0.039
CFH	54.38	127.11	2.34	0.013
CHL1	12.07	25.60	2.12	0.033
COL4A5	2.16	13.30	6.16	0.038
CPE	9.78	75.22	7.69	0.040
CRYAB	229.20	420.80	1.84	0.010
CSGALNACT1	8.22	15.58	1.90	0.020
CSRP1	56.90	128.77	2.26	0.031
CSRP2	10.99	75.74	6.89	0.045
CSRP3	4.79	96.74	20.18	0.004
DBNDD2	21.45	43.61	2.03	0.028
DKK3	41.71	119.77	2.87	0.045
ECSCR	13.94	26.73	1.92	0.007
EDN1	15.45	38.77	2.51	0.031
F3	45.20	131.15	2.90	0.024
FHL3	15.42	33.49	2.17	0.010
FIBIN	11.49	26.57	2.31	0.005
FILIP1	10.64	23.28	2.19	0.030
HEYL	11.33	27.55	2.43	0.048
ID4	20.50	71.28	3.48	0.041
IGFBP3	226.25	481.80	2.13	0.001
ITGA1	19.39	40.52	2.09	0.040
KANK1	27.71	54.33	1.96	0.040
LDB3	0.47	12.10	25.82	0.002
MAFF	12.92	23.78	1.84	0.016
MFAP4	90.59	183.84	2.03	0.010
MMRN1	58.39	141.29	2.42	0.012
MRVI1	8.51	29.28	3.44	0.017
MUSTN1	228.51	505.84	2.21	0.049
NEXN	8.53	21.67	2.54	0.008
NPR3	6.16	13.77	2.23	0.027
NPTX1	7.16	28.10	3.92	0.007
NXN	19.09	34.70	1.82	0.007
PARD6G	8.75	17.92	2.05	0.003
PDE4B	6.14	13.95	2.27	0.004
PDLIM3	20.47	122.74	6.00	0.001
PKHD1L1	3.73	8.44	2.26	0.007
PPP1R14A	21.91	113.95	5.20	0.049
PRELP	140.17	254.74	1.82	0.045
PTPRU	12.63	26.38	2.09	0.009
PTX3	19.38	45.87	2.37	0.008
RCAN2	1.31	17.87	13.64	0.048
RELN	3.80	10.66	2.81	0.025
RRAD	14.23	47.85	3.36	0.021
SIPA1L1	10.39	19.69	1.89	0.015
SYNM	28.29	62.37	2.20	0.022
SYNPO2	40.31	107.85	2.68	0.017
TAGLN	76.87	759.51	9.88	0.047
TGFB3	5.34	17.70	3.32	0.024
THBS4	53.01	147.01	2.77	0.012
TM4SF1	40.44	87.32	2.16	0.022
TMEM150C	11.41	21.94	1.92	0.002
TNC	4.75	86.36	18.18	0.021
TNFAIP8L3	14.04	25.78	1.84	0.011
TSPAN5	19.97	43.12	2.16	0.013

Gene	Mean of TPM value		Ratio	*t*-test
	Subcutaneous fat	Intramuscular fat	Intra/Sub	(*P* vales)
APOBEC2	2.62	12.71	4.86	0.004
BMX	3.38	18.42	5.45	0.039
CLDN11	1.89	11.02	5.84	0.017
CMYA5	3.31	18.89	5.71	0.016
COL4A5	2.16	13.30	6.16	0.038
COL4A6	0.70	5.33	7.57	0.031
CPE	9.78	75.22	7.69	0.040
CSDC2	0.44	2.16	4.86	0.023
CSRP2	10.99	75.74	6.89	0.045
EPHA3	1.22	7.35	6.03	0.030
FABP3	2.50	18.38	7.35	0.009
GGT5	2.06	10.35	5.04	0.021
KCNA5	0.89	5.33	6.01	0.027
NPY1R	0.91	7.36	8.11	0.021
PDLIM3	20.47	122.74	6.00	0.001
PPDPFL	0.97	4.58	4.73	0.011
PPP1R14A	21.91	113.95	5.20	0.049
RCAN2	1.31	17.87	13.64	0.048
SBSPON	7.33	50.50	6.89	0.005
SUSD2	0.50	2.30	4.63	0.030
TAGLN	76.87	759.51	9.88	0.047
TMEM51	5.46	30.64	5.61	0.025
TNC	4.75	86.36	18.18	0.021
TUBA4A	1.46	13.15	9.02	0.007
WFDC1	6.90	28.75	4.17	0.029

Figure S2. List of highly expressed 65 genes and specific 25 genes. The figure show the mean values of TPM and relative to the expression level of Intramuscular fat/ subcutaneous fat (t-test ; four cattle in each sample).

No.	Gene	Name	Entrez Gene ID	Mean of TPM value		Ratio
				Subcutaneous fat	Intramuscular fat	Intra/Sub
1	FABP4	fatty acid binding protein 4	281759	27402	20492	0.75
2	SPARC	secreted protein acidic and cysteine rich	282077	17984	15774	0.88
3	TMSB10	thymosin beta 10	282385	12233	13374	1.09
4	VIM	vimentin	280955	10661	11763	1.10
5	TMSB4X	thymosin beta 4 X-linked	282386	11351	11133	0.98
6	COX3	cytochrome c oxidase subunit III	3283883	10847	11087	1.02
7	ACTB	actin beta	280979	7507	8700	1.16
8	CLU	clusterin	280750	8033	8051	1.00
9	SCD	stearoyl-CoA desaturase	280924	9056	7856	0.87
10	COX1	cytochrome c oxidase subunit I	3283879	7122	6656	0.93
11	ITM2B	integral membrane protein 2B	510575	6541	5708	0.87
12	ADIPOQ	adiponectin	282865	6000	5253	0.88
13	ATP6	ATP synthase F0 subunit 6	3283882	4987	5188	1.04
14	ANXA2	annexin A2	282689	4848	4512	0.93
15	CIDEC	cell death inducing DFFA like effector c	534607	5447	4180	0.77

Figure S3. List of top 15 genes with high expression of intramuscular fat and subcutaneous fat. The figure show the mean values of TPM and relative to the expression level of Intramuscular fat/ subcutaneous fat.

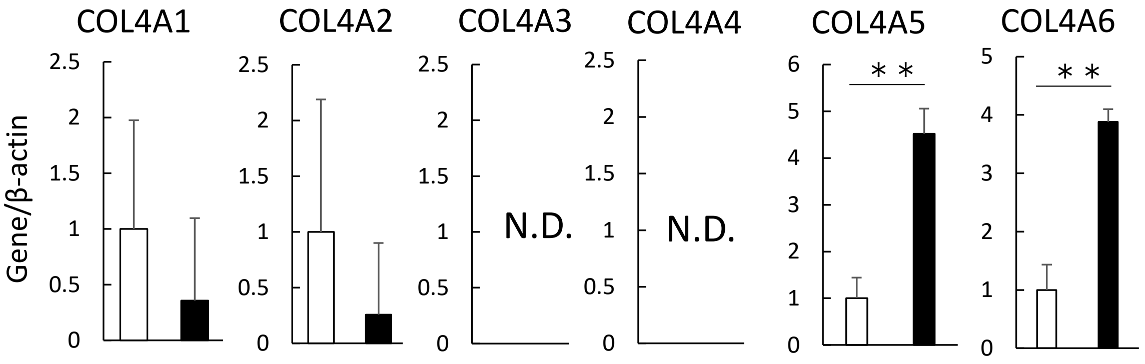

Figure S4: Expression analysis of COL4A isoforms by qPCR. The graph shows the relative values compared by qPCR. The graphs show the mean values with a significant difference (*t*-test; ** $p < 0.01$, 3 cattle in each sample, ± standard error.)

ID	Gene Name
281730	C-terminal binding protein 2(CTBP2)
505453	CREB binding protein(CREBBP)
512841	CRK proto-oncogene, adaptor protein(CRK)
281788	G protein subunit alpha 11(GNA11)
281791	G protein subunit alpha i2(GNAI2)
536654	G protein subunit alpha q(GNAQ)
281201	G protein subunit beta 1(GNB1)
613930	G protein subunit gamma 10(GNG10)
511812	G protein subunit gamma 11(GNG11)
287018	G protein subunit gamma 5(GNG5)
540378	MDM2 proto-oncogene(MDM2)
280855	MET proto-oncogene, receptor tyrosine kinase(MET)
538477	RAS like proto-oncogene A(RALA)
505238	RAS like proto-oncogene B(RALB)
508233	RELA proto-oncogene, NF-kB subunit(RELA)
538628	RUNX1 translocation partner 1(RUNX1T1)
510276	Ras association domain family member 1(RASSF1)
785911	Rho associated coiled-coil containing protein kinase 1(ROCK1)
282041	Rho associated coiled-coil containing protein kinase 2(ROCK2)
516010	SMAD family member 2(SMAD2)
512355	TNF receptor associated factor 2(TRAF2)
517850	adaptor protein, phosphotyrosine interacting with PH domain and leucine zipper 1(APPL1)
509936	adenylate cyclase 6(ADCY6)
281010	aryl hydrocarbon receptor nuclear translocator(ARNT)
514386	baculoviral IAP repeat containing 3(BIRC3)
539003	catenin beta 1(CTNNB1)
511602	collagen type IV alpha 5 chain(COL4A5)
535219	cullin 2(CUL2)
510618	cyclin dependent kinase 4(CDK4)
445417	frizzled class receptor 1(FZD1)
445418	frizzled class receptor 6(FZD6)
404126	histone deacetylase 1(HDAC1)
281876	integrin subunit beta 1(ITGB1)
782518	lysophosphatidic acid receptor 6(LPAR6)
407219	melanogenesis associated transcription factor(MITF)
616744	mutS homolog 3(MSH3)
540526	mutS homolog 6(MSH6)
616115	nuclear factor kappa B subunit 1(NFKB1)
526392	nuclear factor kappa B subunit 2(NFKB2)
281985	phospholipase C beta 4(PLCB4)
281987	phospholipase C gamma 1(PLCG1)
282331	prostaglandin E receptor 4(PTGER4)
506270	protein tyrosine kinase 2(PTK2)
534280	retinoic acid receptor alpha(RARA)
504943	retinoid X receptor beta(RXRB)
518880	ring-box 1(RBX1)
534069	transforming growth factor beta 2(TGFB2)
538957	transforming growth factor beta 3(TGFB3)
282382	transforming growth factor beta receptor 1(TGFBR1)
497019	tropomyosin 3(TPM3)

Figure S5: List of pathway analyses.

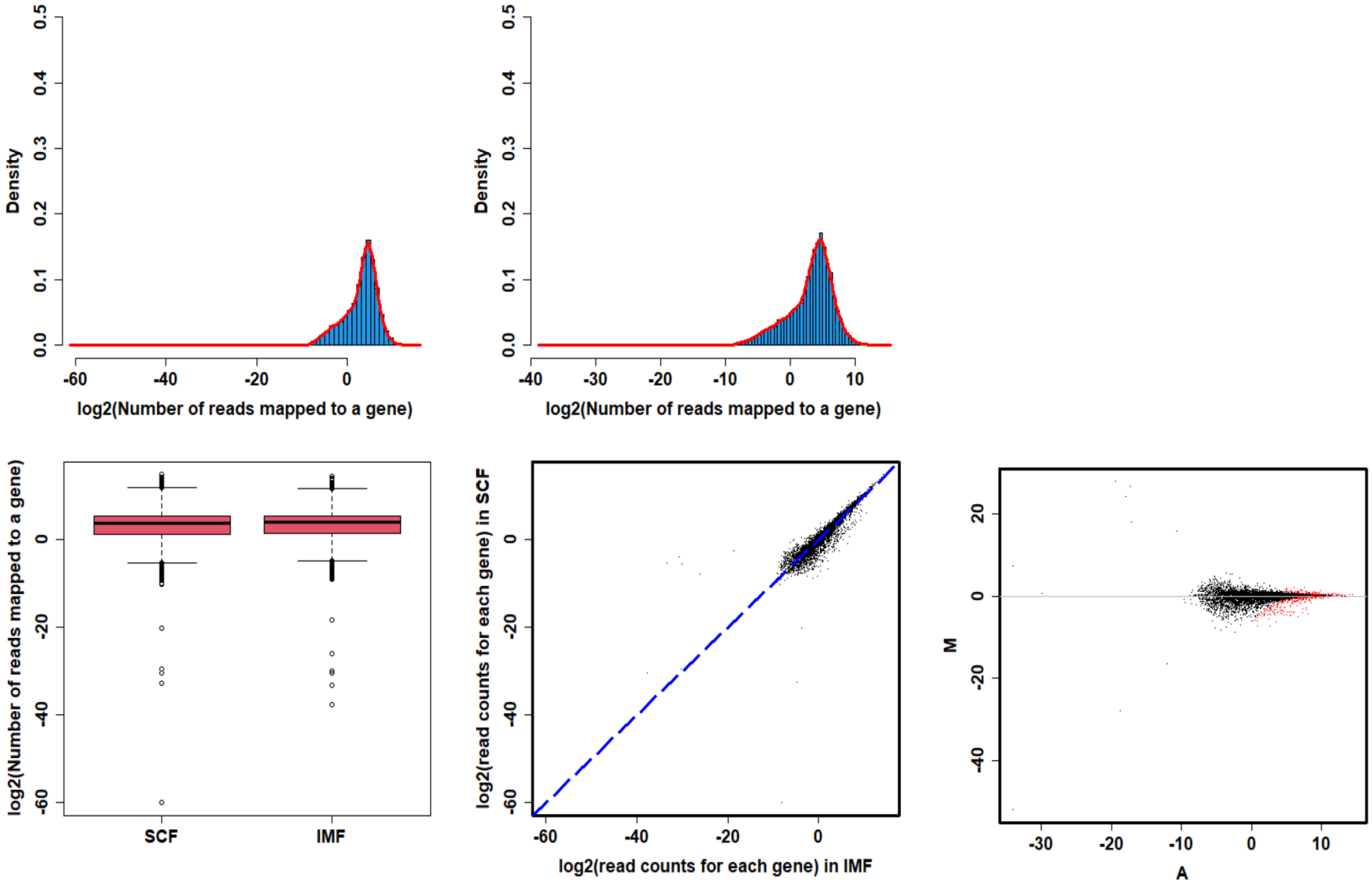

Figure S6: Comparison between two groups by DEGseq2 package.
